# Supplementary material for: Pluripotency reprogramming by competent and incompetent POU factors uncovers temporal dependency for Oct4 and Sox2
Source: Nat Commun. 2019 Aug 2;10:3477. doi: 10.1038/s41467-019-11054-7 (PMC6677745; doi:10.1038/s41467-019-11054-7)
Supplement: Supplementary file 1 — Supplementary Information [file 41467_2019_11054_MOESM1_ESM.pdf]

Supplementary Information

Pluripotency reprogramming by competent and incompetent POU factors uncovers temporal dependency for Oct4 and Sox2

Malik et al.

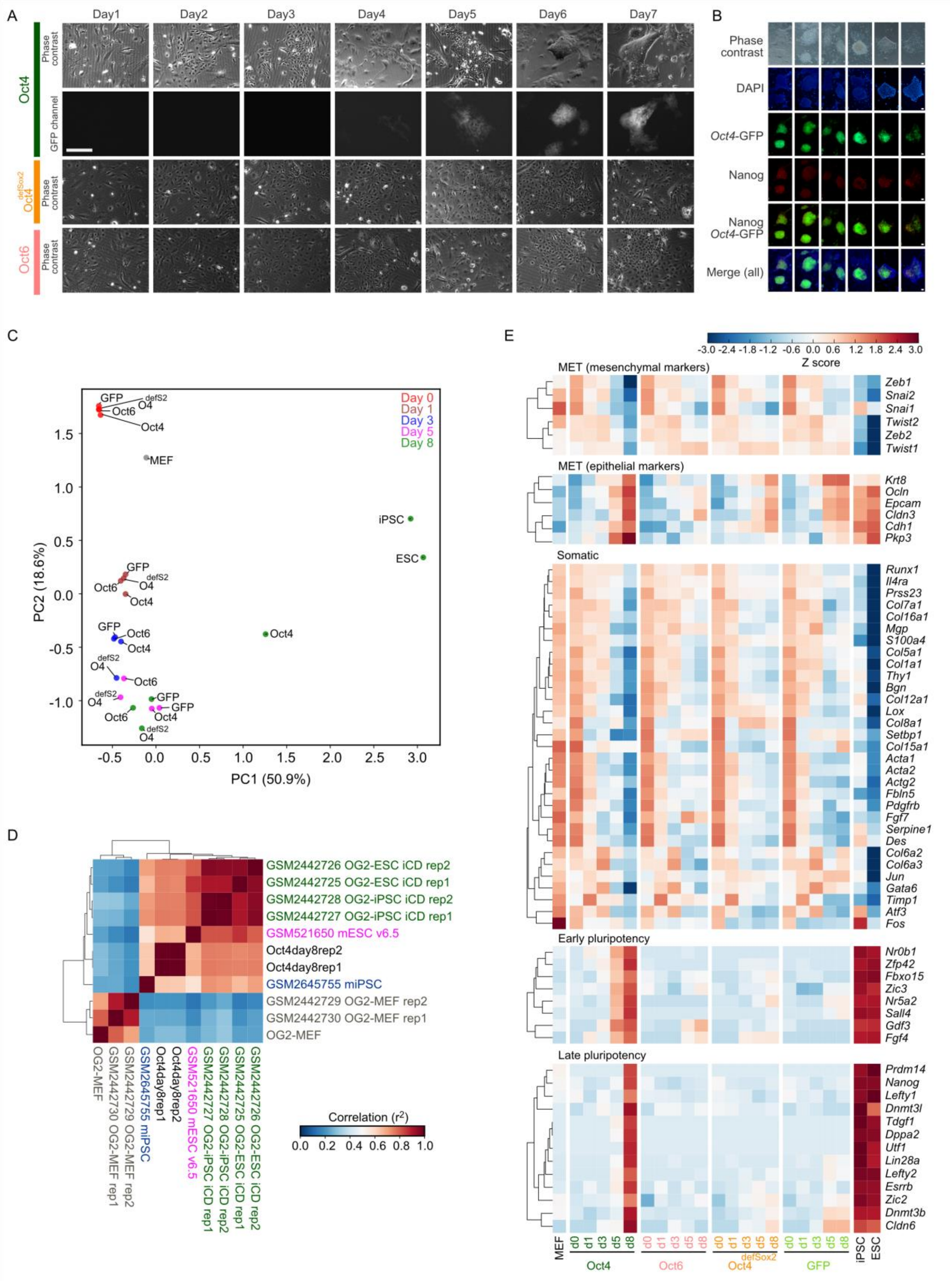
A
Day1
Day2
Day3
Day4
Day5
Day6
Day7
Oct4
Phase contrast
GFP channel
Oct4 defSox2
Oct6
B
Phase contrast
DAPI
Oct4-GFP
Nanog
Nanog Oct4-GFP
Merge (all)
C
Day 0
Day 1
Day 3
Day 5
Day 8
GFP
Oct6
Oct4
O4 defS2
MEF
iPSC
ESC
PC2 (18.6%)
PC1 (50.9%)
D
GSM2442726 OG2-ESC iCD rep2
GSM2442725 OG2-ESC iCD rep1
GSM2442728 OG2-iPSC iCD rep2
GSM2442727 OG2-iPSC iCD rep1
GSM521650 mESC v6.5
Oct4day8rep2
Oct4day8rep1
GSM2645755 miPSC
GSM2442729 OG2-MEF rep2
GSM2442730 OG2-MEF rep1
OG2-MEF
Correlation (r2)
E
Z score
MET (mesenchymal markers)
Zeb1
Snai2
Snai1
Twist2
Zeb2
Twist1
MET (epithelial markers)
Krt8
Ocln
Epcam
Cldn3
Cdh1
Pkp3
Somatic
Runx1
Il4ra
Prss23
Col7a1
Col16a1
Mgp
S100a4
Col5a1
Col1a1
Thy1
Bgn
Col12a1
Lox
Col8a1
Setbp1
Col15a1
Acta1
Acta2
Actg2
Fbln5
Pdgfrb
Fgf7
Serpine1
Des
Col6a2
Col6a3
Jun
Gata6
Timp1
Atf3
Fos
Early pluripotency
Nr0b1
Zfp42
Fbxo15
Zic3
Nr5a2
Sall4
Gdf3
Fgf4
Late pluripotency
Prdm14
Nanog
Lefty1
Dnmt3l
Tdgf1
Dppa2
Utf1
Lin28a
Lefty2
Esrrb
Zic2
Dnmt3b
Cldn6
MEF
d0
d1
d3
d5
d8
iPSC
ESC
Oct4
Oct6
Oct4 defSox2
GFP

Supplementary Figure 1. **Oct4 is critical for the induction of pluripotency genes at late stage of reprogramming, Related to Figure 1.**

(A) Representative microscopy photographs of cells expressing Oct4-SK, Oct6-SK or $Oct4^{defSox2}$-SK in iCD1 medium taken with phase contrast or GFP (*Oct4*-GFP reporter) channels at indicated days; 10x objective, scale 200 μm.

(B) Fluorescence images of *Oct4*-GFP signals and Nanog immunostaining at day 8 of reprogramming in Oct4-SK cells; 10x objective, scale 80 μm. The antibodies are in Supplementary Table 11.

(C) Principle component analysis for the RNA-seq gene expression data. Publically available bulk RNA-seq data for iPSC and ESCs[1] are shown as reference.

(D) Hierarchically clustered heatmap based on r2 correlation coefficients using MEFs and day 8 RNA-seq signals from this study and published bulk ESCs and iPSC cultured in chemically defined or serum/LIF conditions. Prefix in sample name are GEO accession numbers. Culture conditions for these samples are listed in Supplementary Table 1.

(E) Heatmaps show the expression dynamics of selected markers genes for the indicated categories across four reprogramming cocktails. Data are presented as row-wise Z-score.

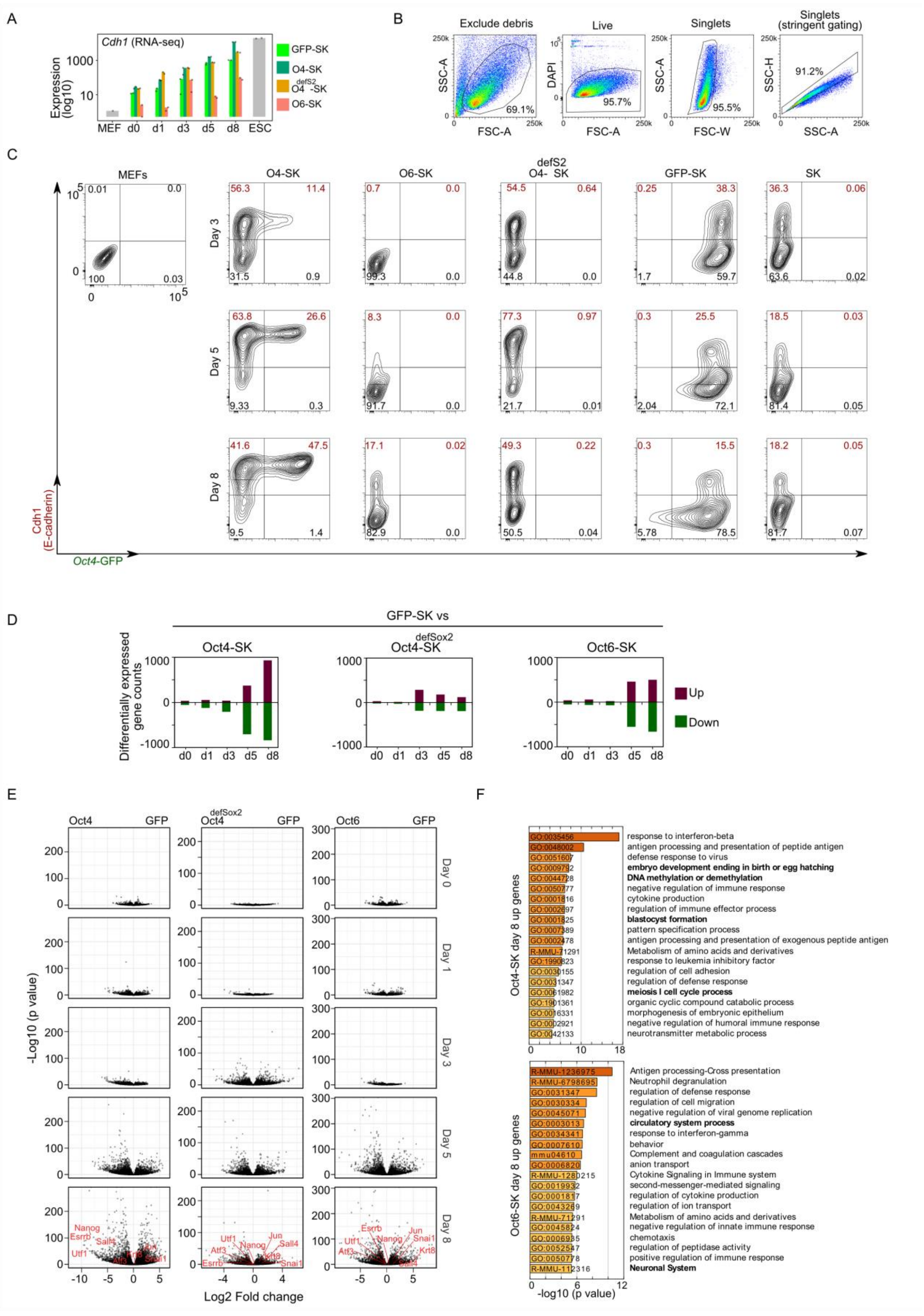
A
Cdh1 (RNA-seq)
Expression (log10)
1000
10
MEF
d0
d1
d3
d5
d8
ESC
GFP-SK
O4-SK
O4 defS2 -SK
O6-SK
B
Exclude debris
Live
Singlets
Singlets (stringent gating)
SSC-A
FSC-A
DAPI
FSC-W
SSC-H
69.1%
95.7%
95.5%
91.2%
C
MEFs
O4-SK
O6-SK
defS2 O4- SK
GFP-SK
SK
Day 3
Day 5
Day 8
Cdh1 (E-cadherin)
Oct4-GFP
D
GFP-SK vs
Oct4-SK
defSox2 Oct4-SK
Oct6-SK
Differentially expressed gene counts
Up
Down
E
Oct4
GFP
defSox2 Oct4
Oct6
Day 0
Day 1
Day 3
Day 5
Day 8
-Log10 (p value)
Log2 Fold change
Nanog
Esrrb
Sall4
Utf1
Jun
Atf3
Snai1
Krt8
F
Oct4-SK day 8 up genes
response to interferon-beta
antigen processing and presentation of peptide antigen
defense response to virus
embryo development ending in birth or egg hatching
DNA methylation or demethylation
negative regulation of immune response
cytokine production
regulation of immune effector process
blastocyst formation
pattern specification process
antigen processing and presentation of exogenous peptide antigen
Metabolism of amino acids and derivatives
response to leukemia inhibitory factor
regulation of cell adhesion
regulation of defense response
meiosis I cell cycle process
organic cyclic compound catabolic process
morphogenesis of embryonic epithelium
negative regulation of humoral immune response
neurotransmitter metabolic process
Oct6-SK day 8 up genes
Antigen processing-Cross presentation
Neutrophil degranulation
regulation of defense response
regulation of cell migration
negative regulation of viral genome replication
circulatory system process
response to interferon-gamma
behavior
Complement and coagulation cascades
anion transport
Cytokine Signaling in Immune system
second-messenger-mediated signaling
regulation of cytokine production
regulation of ion transport
Metabolism of amino acids and derivatives
negative regulation of innate immune response
chemotaxis
regulation of peptidase activity
positive regulation of immune response
Neuronal System
-log10 (p value)

Supplementary Figure 2. **The Oct6-SK condition fails to activate the epithelial program**. **Related to Figure 1.**

(A) Gene expression (mean tag counts as bar and individual technical replicate as dots) for *Cdh1* (encoding Cadherin-1/E-cadherin) during reprogramming.

(B) Gating strategy for the FACS analysis. FSC=forward-scatter; SSC: side-scatter; A: area; H: height; W: width.

(C) Flow cytometry analysis using the *Oct4*-GFP (OG2) signal and Cadherin-1 expression using reprogramming cells at days 3, 5 and 8 expressing Oct4-SK, Oct6-SK, $Oct4^{defSox2}$ –SK, GFP-SK or SK cocktails. For this experiment, $5.5x10^4$ OG2-MEFs were plated per well of a 6-well plate. Percentage of cells positive or negative for Cadherin-1 and *Oct4*-GFP reporter are indicated in each quadrant. Source data for (B) and (C) are provided as a Source Data file.

(D) The number of differentially expressed (DE) genes at different days of reprogramming is shown as bar plots in GFP-SK versus the three indicated conditions.

(E) Volcano plots of differential gene expression with respect to GFP-SK at indicated days of reprogramming are shown for Oct4-SK (left panel), $Oct4^{defSox2}$-SK (middle) and Oct6-SK (right) conditions. Selected somatic and pluripotency marker genes are labelled at day 8. Differentially expressed gene list is provided in Supplementary Data 1.

(F) GO analysis performed using metascape (http://metascape.org) of genes differentially upregulated (fold change >2) in GFP-SK vs Oct4-SK (upper) and GFP-SK vs Oct6-SK (lower) at day 8.

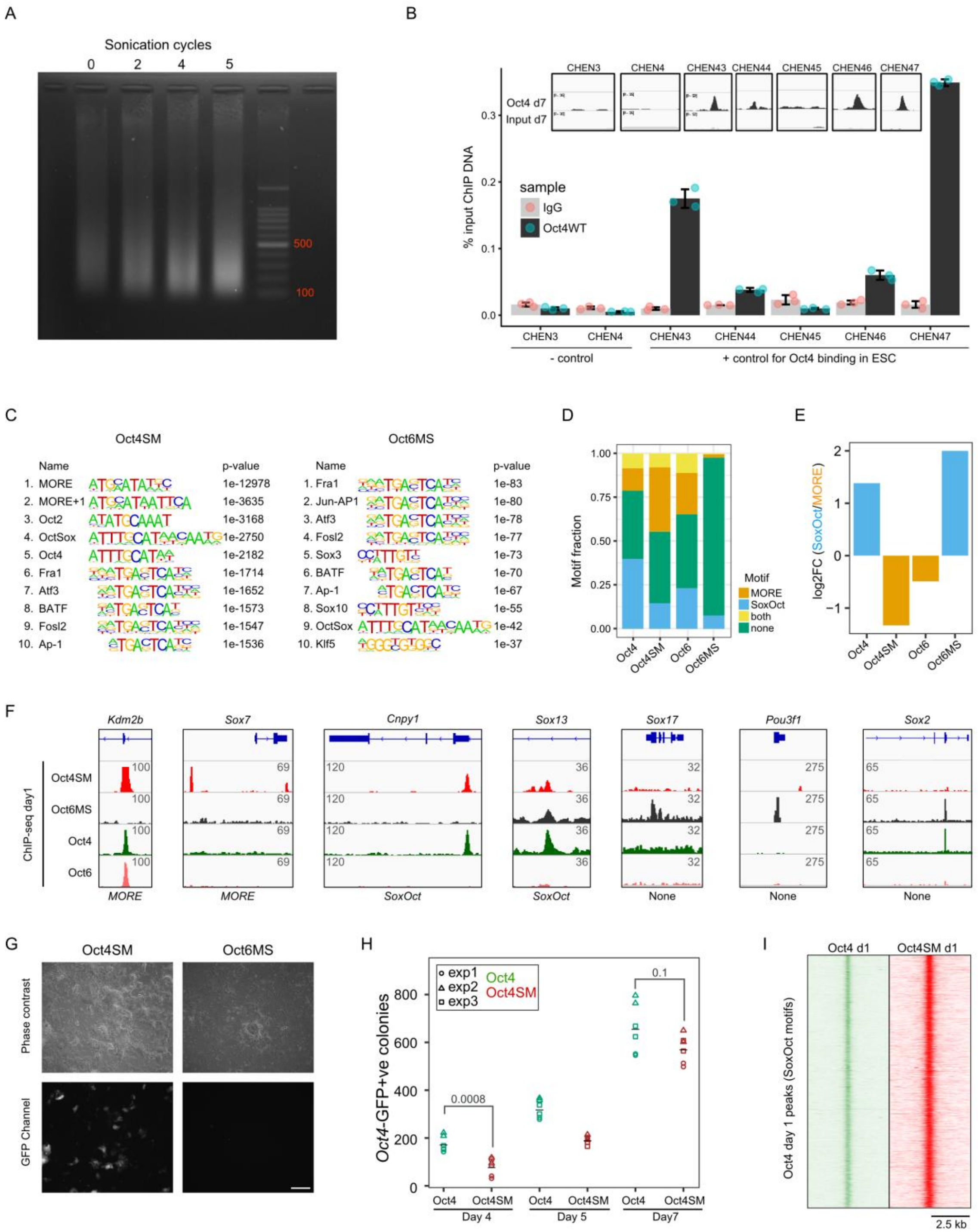

A
Sonication cycles
0 2 4 5
500
100
B
Oct4 d7
Input d7
CHEN3 CHEN4 CHEN43 CHEN44 CHEN45 CHEN46 CHEN47
% input ChIP DNA
sample
IgG
Oct4WT
- control
+ control for Oct4 binding in ESC
C
Oct4SM
Name p-value
1. MORE 1e-12978
2. MORE+1 1e-3635
3. Oct2 1e-3168
4. OctSox 1e-2750
5. Oct4 1e-2182
6. Fra1 1e-1714
7. Atf3 1e-1652
8. BATF 1e-1573
9. Fosl2 1e-1547
10. Ap-1 1e-1536
Oct6MS
Name p-value
1. Fra1 1e-83
2. Jun-AP1 1e-80
3. Atf3 1e-78
4. Fosl2 1e-77
5. Sox3 1e-73
6. BATF 1e-70
7. Ap-1 1e-67
8. Sox10 1e-55
9. OctSox 1e-42
10. Klf5 1e-37
D
Motif fraction
Motif
MORE
SoxOct
both
none
Oct4 Oct4SM Oct6 Oct6MS
E
log2FC (SoxOct/MORE)
Oct4 Oct4SM Oct6 Oct6MS
F
Kdm2b Sox7 Cnpy1 Sox13 Sox17 Pou3f1 Sox2
ChIP-seq day1
Oct4SM
Oct6MS
Oct4
Oct6
MORE MORE SoxOct SoxOct None None None
G
Oct4SM Oct6MS
Phase contrast
GFP Channel
H
exp1
exp2
exp3
Oct4
Oct4SM
Oct4-GFP+ve colonies
0.0008
0.1
Oct4 Oct4SM Oct4 Oct4SM Oct4 Oct4SM
Day 4 Day 5 Day7
I
Oct4 d1 Oct4SM d1
Oct4 day 1 peaks (SoxOct motifs)
2.5 kb

Supplementary Figure 3. Point mutations switch motif binding preferences for POU factors, Related to Figure 2

(A) Sonication settings to obtain 100-500 bp DNA fragments for ChIP-sequencing. DNA marker sizes are indicated and lanes are labelled with sonication cycle numbers (Covaris S220 Focused-Ultrasonicator).

(B) ChIP-qPCR at loci reported previously[2], using the Oct4 antibody at day 7 of reprogramming. Genome browser tracks for day 7 Oct4 ChIP-seq and input signals are shown on top (ChIP-qPCR primers are listed in Supplementary Table 3). Dots represent individual data points (n = 3, technical replicates) and error bars are (mean +/- sd).

(C) Top 10 homer motifs with matches to known motifs and corresponding p-values determined at day 1 ChIP-seq peaks for Oct4^{S151M} and Oct6^{M151S}. Motifs were re-named from the HOMER database: Pit1 to *MORE*, Pit1+1bp to *MORE*+1.

(D) Fraction of locations containing *MORE* (including *MORE* variants with 0 or 1 bp spacers) or *SoxOct* elements at different reprogramming stages for indicated wild-type and mutant POU factors. 'Both' refers to peaks where motif scanning detected *MORE* and *SoxOct* motifs concurrently and 'none' the absence of either of the two motifs.

(E) Counts for matches to *SoxOct* and *MORE* motifs in (D) are represented as log2 ratios.

(F) Genome browser tracks of representative location for day 1 ChIP-seq from indicated conditions for loci with different motifs. Genomic coordinates for the summits are given in Supplementary Table 4.

(G) Representative phase contrast and *Oct4*-GFP fluorescence images of cells reprogrammed with Oct4^{S151M}-SK and Oct6^{M151S}-SK conditions (labelled as Oct4SM and Oct6MS in the figures) at day 8 post transduction. 5x objective, scale 500 µm.

(H) *Oct4*-GFP positive colony counts are shown for three independent biological replicates each consisting of two technical replicates (n = 6) at days 4, 5 and 7. Student's t-test (two tailed, unpaired, with assumption of equal variance) was performed to assess significance.

(I) ChIP-seq signal heatmap for Oct4-SK and Oct4^{S151M}-SK conditions at day 1 of reprogramming. Rows are Oct4 peaks containing *SoxOct* motifs.

Source data are provided as a Source Data file for (B) and (H).

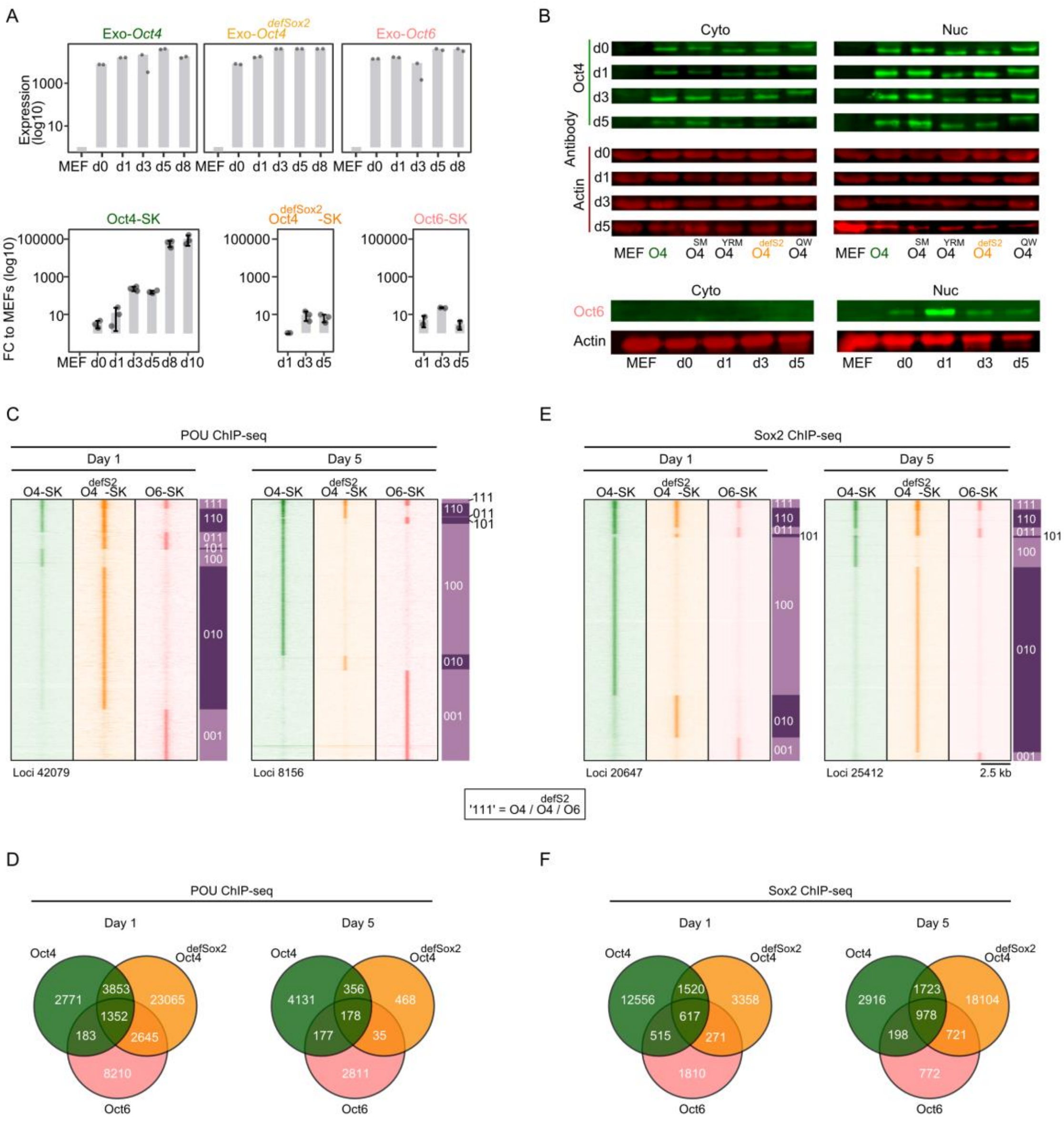
A
Exo-Oct4
Exo-Oct4 defSox2
Exo-Oct6
Expression (log10)
1000
10
MEF d0 d1 d3 d5 d8
Oct4-SK
Oct4 defSox2 -SK
Oct6-SK
endo-Pou5f1 FC to MEFs (log10)
100000
1000
10
MEF d0 d1 d3 d5 d8 d10
d1 d3 d5
B
Cyto
Nuc
Oct4
Antibody
Actin
d0
d1
d3
d5
MEF O4 SM O4 YRM O4 defS2 O4 QW O4
Oct6
Actin
MEF d0 d1 d3 d5
C
POU ChIP-seq
Day 1
Day 5
O4-SK
O4 defS2 -SK
O6-SK
111
110
011
101
100
010
001
Loci 42079
Loci 8156
E
Sox2 ChIP-seq
Loci 20647
Loci 25412
2.5 kb
'111' = O4 / O4 defS2 / O6
D
POU ChIP-seq
Day 1
Day 5
Oct4
Oct4 defSox2
Oct6
2771
3853
23065
1352
183
2645
8210
4131
356
468
178
177
35
2811
F
Sox2 ChIP-seq
12556
1520
3358
617
515
271
1810
2916
1723
18104
978
198
721
772

Supplementary Figure 4. Reprogramming incompetent POUs are re-distributed to disparate binding sites, Related to Figure 3

(A) Gene expression of total *Oct4* (from Oct4-SK and $Oct4^{defSox2}$-SK expressing cells) and total *Oct6* (from Oct6-SK expressing cells) shown as mean normalized tag counts from replicate RNAseq experiments (upper panel, individual technical replicate as dots). The lower panels show the expression of endogenous *Oct4* in the three conditions determined by qRT-PCR (mean +/- sd; n = 3 biological replicates). qRT-PCR primers are listed in Supplementary Table 9.

(B) Western blots showing distribution of POU proteins in cytoplasmic (Cyto) and nuclear (Nuc) fractions at indicated days of reprogramming. Actin was used as housekeeping protein control. O4SM corresponds to the $Oct4^{S151M}$ protein (Supplementary Figures 3C-I) and $O4^{YRM}$ and $O4^{QW}$ are Oct4 mutants not further discussed in this manuscript but were analysed in the same Western blot.

(C) Heatmap of intersections of ChIP-seq peaks for POU factors in indicated conditions at days 1 and 5 of reprogramming. Rows are the union of POU peak summits in Oct4-SK, $Oct4^{defSox2}$-SK and Oct6-SK conditions at days 1 and 5 of reprogramming arranged in 7 binary occupancy trajectories. 1 indicates presence and 0 absence of binding; digits from left to right are Oct4-SK, $Oct4^{defSox2}$-SK and Oct6-SK conditions. Adjacent summits were merged if they were within 400bp. ChIP-seq signals are shown in 5 kb window centred at peak summits.

(D) Venn diagrams of ChIP-seq peaks overlap of POU factors of this study at day 1 (left) and day 5 (right).

(E) Same as (C) but done for Sox2 ChIP-seq in Oct4-SK, Oct6-SK and $Oct4^{defSox2}$-SK conditions at days 1 and 5 of reprogramming.

(F) Similar to (E) but here Venn diagrams represent Sox2 ChIP-seq peaks intersections at day 1 (left) and day 5 (right) of reprogramming.

Genomic locations for (C) and (E) are provided in Supplementary Data 2. Source data for (A, lower panel) and (B) are provided as a Source Data file.

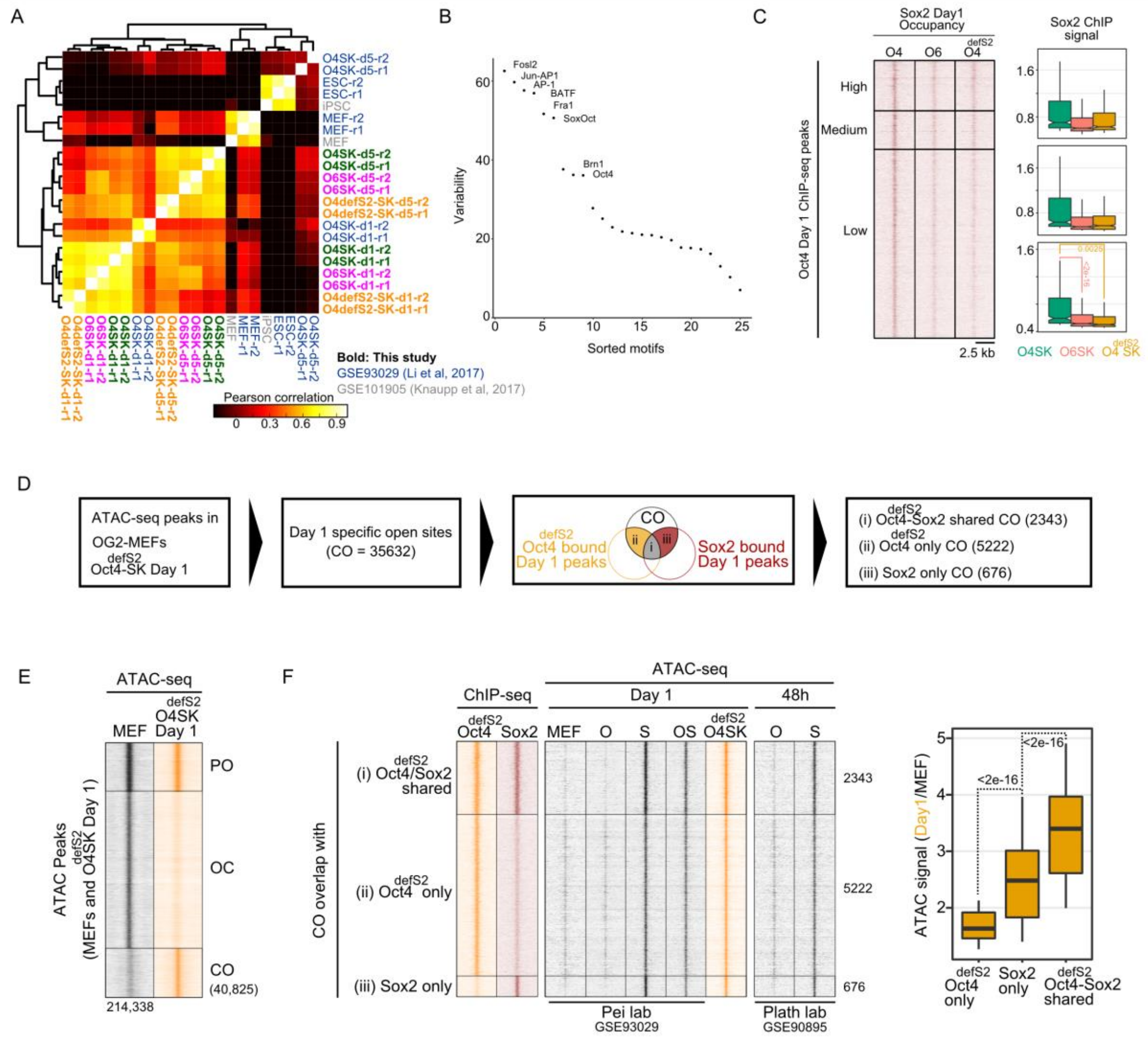

A
B
C
D
E
F
Bold: This study
GSE93029 (Li et al, 2017)
GSE101905 (Knaupp et al, 2017)
Pearson correlation
Variability
Sorted motifs
Fosl2
Jun-AP1
AP-1
BATF
Fra1
SoxOct
Brn1
Oct4
Sox2 Day1 Occupancy
Sox2 ChIP signal
Oct4 Day 1 ChIP-seq peaks
High
Medium
Low
2.5 kb
O4SK
O6SK
O4 defS2 SK
ATAC-seq peaks in OG2-MEFs Oct4defS2-SK Day 1
Day 1 specific open sites (CO = 35632)
Oct4defS2 bound Day 1 peaks
Sox2 bound Day 1 peaks
(i) Oct4defS2-Sox2 shared CO (2343)
(ii) Oct4defS2 only CO (5222)
(iii) Sox2 only CO (676)
ATAC-seq
MEF
O4defS2SK Day 1
PO
OC
CO
(40,825)
214,338
ATAC Peaks (MEFs and O4defS2SK Day 1)
ChIP-seq
Day 1
48h
(i) Oct4defS2/Sox2 shared
(ii) Oct4defS2 only
(iii) Sox2 only
CO overlap with
2343
5222
676
Pei lab
GSE93029
Plath lab
GSE90895
ATAC signal (Day1/MEF)
<2e-16
<2e-16
Oct4defS2 only
Sox2 only
Oct4defS2-Sox2 shared

Supplementary Figure 5. Chromatin dynamics in the 'Oct4^{defSox2} –SK condition, Related to Figure 4

(A) Hierarchical clustering of the pairwise correlation coefficients (R^2) of Oct4-SK, Oct4^{defSox2}-SK and Oct6-SK ATAC-seq peaks from this study (bold font) as well as publicly available ATAC-seq data from Knaupp et al (grey) and Li et al (blue) [1,3]. r1 and r2 denote replicates.

(B) Ranked accessibility variation determined by chromVAR for day 1 and 5 ATAC-seq peaks of the three conditions (Oct4-SK, Oct4^{defSox2}-SK and Oct6-SK) marked by a selection of motifs taken from the HOMER database with high variability.

(C) Sox2 ChIP-seq signal from three POU-SK conditions are plotted as heatmap and boxplots on day 1 Oct4 ChIP-seq peaks grouped by their accessibility in MEFs.

(D) Analysis flow chart for defining the closed-to-open (CO) location using MEF and day 1 Oct4^{defSox2}-SK ATAC-seq peaks followed by grouping for Oct4^{defSox2}/Sox2 day 1 binding.

(E) ATAC-seq signal heatmaps for the three accessibility trajectories.

(F) The CO category from (F) was grouped by ChIP-seq peaks of Oct4^{defSox2} and Sox2 (in Oct4^{defSox2}SK) at day 1. ChIP-seq and ATAC-seq signal heatmaps are shown for these groups at the indicated conditions. Boxplots represent Oct4^{defSox2}-SK/MEF ATAC-seq signal ratios at the three peak sets. The ATAC-seq samples for MEFs and day 1 (O, S, OS) were downloaded from GSE93029[1] and at 48 hours (O, S) from GSE90895[4].

ATAC-seq and Sox2 ChIP-seq signals were normalized by EASeq (DNA fragments per kilobase pairs (kbp) per million (M) reads) and POU ChIP-seq signals were quantile normalized. P-values in (C, F), were calculated using pairwise comparisons with the unpaired Wilcoxon rank sum test (R function pairwise.wilcox.test) and adjusted for multiple testing using the Holm method. Genomic locations for (E, F) are provided in Supplementary Data 2. For the boxplots, the midline indicates the median, boxes indicate the upper and lower quartiles and the whiskers indicate 1.5 times interquartile range.

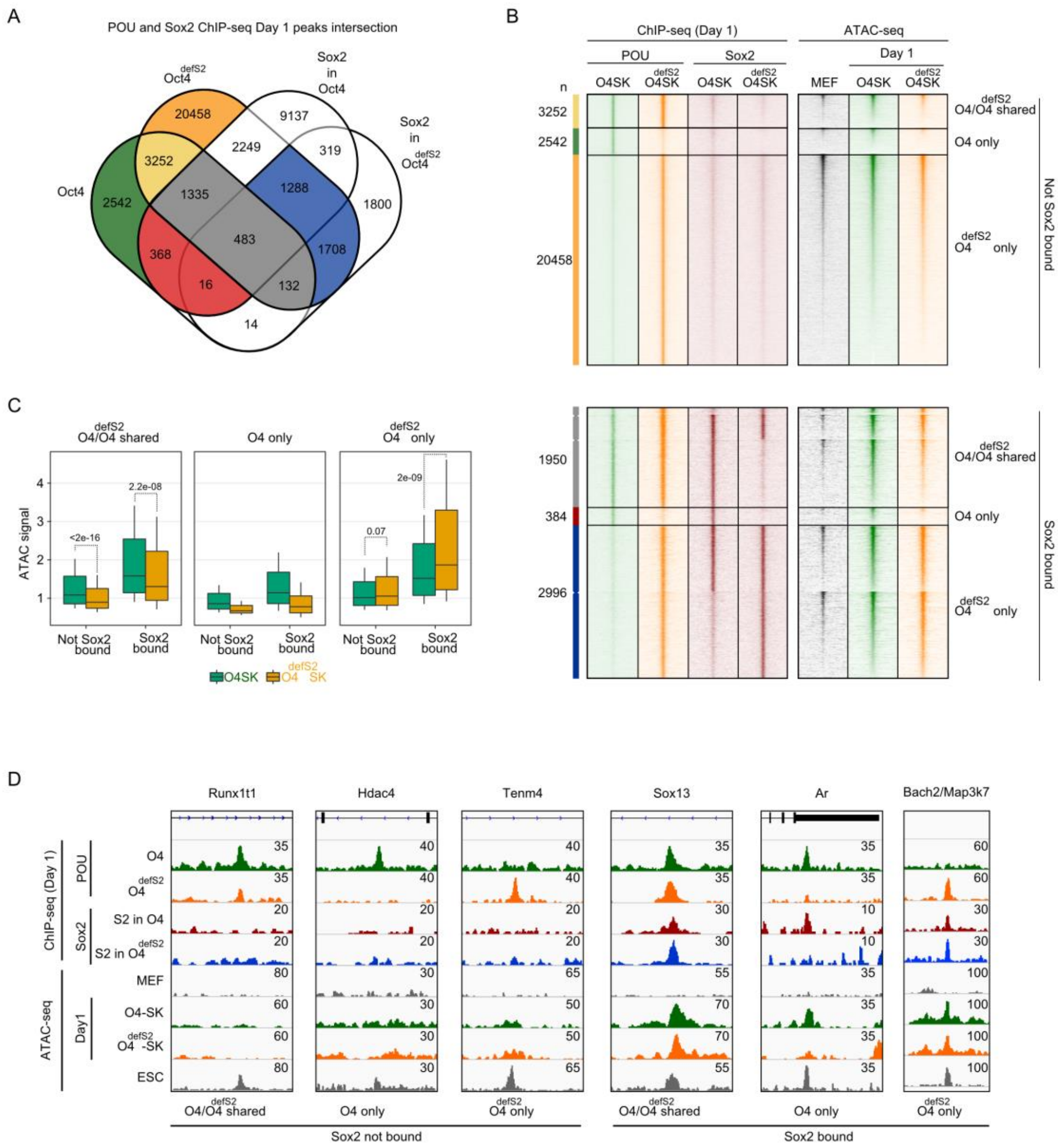
A
POU and Sox2 ChIP-seq Day 1 peaks intersection
Oct4 defS2
Sox2 in Oct4
Sox2 in Oct4 defS2
Oct4
20458
9137
2249
319
3252
1335
1288
1800
2542
483
1708
368
16
132
14
B
ChIP-seq (Day 1)
ATAC-seq
POU
Sox2
Day 1
n
O4SK
defS2 O4SK
MEF
3252
2542
20458
1950
384
2996
O4/O4 defS2 shared
O4 only
O4 defS2 only
Not Sox2 bound
Sox2 bound
C
ATAC signal
<2e-16
2.2e-08
0.07
2e-09
Not Sox2 bound
Sox2 bound
O4SK
O4 defS2 SK
D
Runx1t1
Hdac4
Tenm4
Sox13
Ar
Bach2/Map3k7
ChIP-seq (Day 1)
POU
Sox2
O4
O4 defS2
S2 in O4
S2 in O4 defS2
ATAC-seq
Day1
MEF
O4-SK
O4 defS2 -SK
ESC
Sox2 not bound
Sox2 bound

Supplementary Figure 6. Oct4^{defSox2} interfere with Sox2 mediated chromatin opening, Related to Figure 4

(A) ChIP-seq peaks at day 1 for Oct4, Oct4^{defSox2}, Sox2 in Oct4 and Sox2 in Oct4^{defSox2} conditions were intersected and peak numbers are represented as Venn diagram. Six main binding site categories are highlighted with six different colours.

(B) ChIP-seq peaks for Oct4, Oct4^{defSox2} were intersected to define common and specific sites further grouped by the absence (upper heatmaps) or presence of Sox2 binding (lower heatmaps). ChIP-seq and ATAC-seq signals are visualised as heatmaps over these binding categories for the indicated conditions. The MEFs sample in grey is from GSE93029[1]. Bars to the left are color coded as the six categories in the Venn diagram in (A).

(C) Boxplot for comparing the ATAC-seq signals in (B) for common and specific Oct4/Oct4^{defSox2} peaks in the absence or presence of Sox2. For the boxplots, the midline indicates the median, boxes indicate the upper and lower quartiles and the whiskers indicate 1.5 times interquartile range.

(D) Genome browser tracks showing examples for the six binding categories.

P-values in (C) were calculated using the unpaired Wilcoxon rank sum test (R function pairwise.wilcox.test) and adjusted for multiple testing using the Holm method. ATAC-seq boxplots show signals normalised using EAseq (DNA fragments per kilobase pairs (kbp) per million (M) reads) Genomic locations for (B) are provided in Supplementary Data 2 and coordinates for ChIP-seq peaks in (D) are listed in Supplementary Table 4.

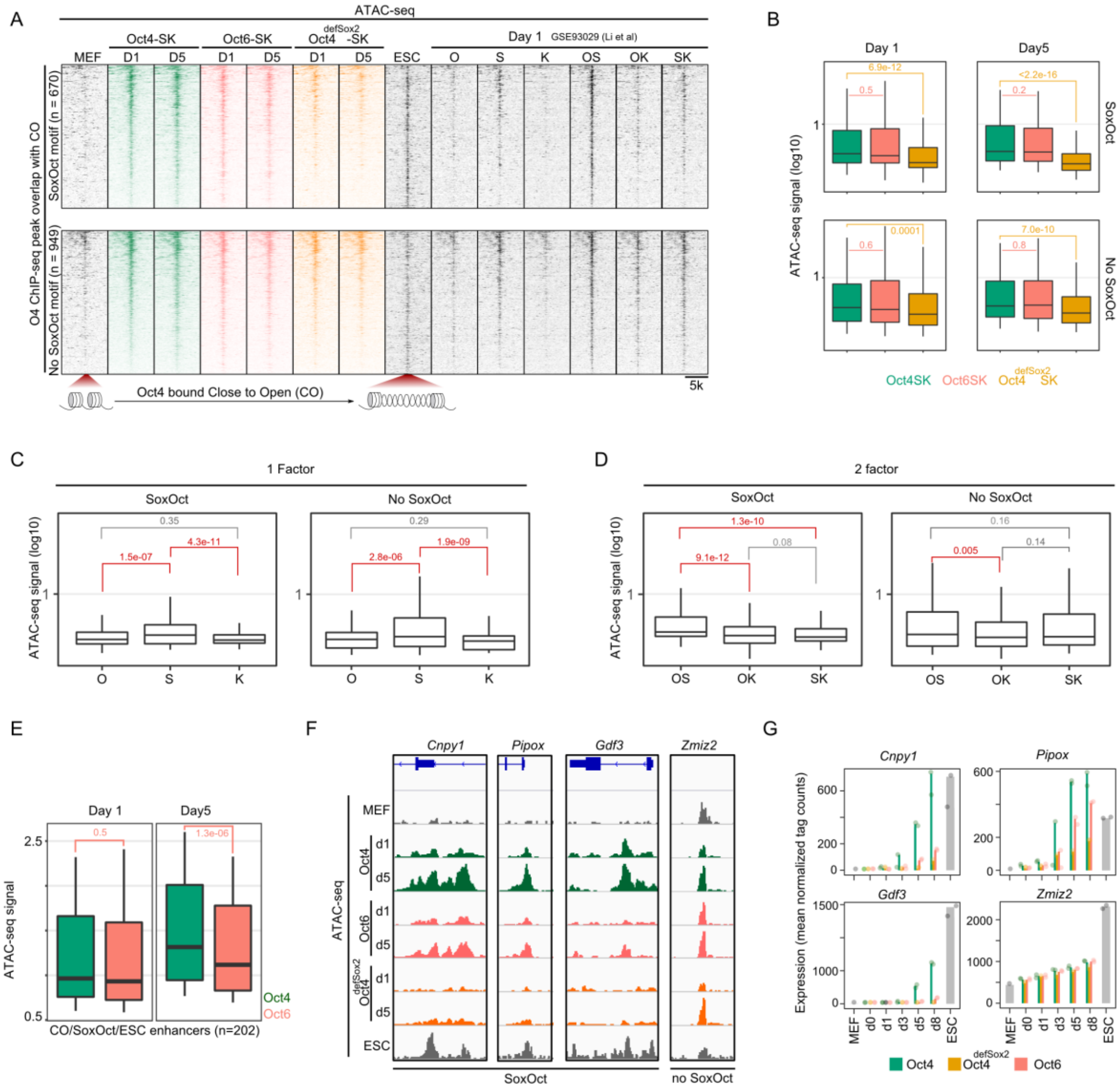
A
ATAC-seq
MEF
Oct4-SK
Oct6-SK
defSox2
Oct4 -SK
ESC
Day 1 GSE93029 (Li et al)
D1
D5
O
S
K
OS
OK
SK
O4 ChIP-seq peak overlap with CO
SoxOct motif (n = 670)
No SoxOct motif (n = 949)
Oct4 bound Close to Open (CO)
5k
B
Day 1
Day5
ATAC-seq signal (log10)
SoxOct
No SoxOct
Oct4SK Oct6SK Oct4 SK
C
1 Factor
SoxOct
No SoxOct
ATAC-seq signal (log10)
D
2 factor
E
Day 1
Day5
ATAC-seq signal
CO/SoxOct/ESC enhancers (n=202)
Oct4
Oct6
F
Cnpy1
Pipox
Gdf3
Zmiz2
ATAC-seq
SoxOct
no SoxOct
G
Expression (mean normalized tag counts)
MEF
d0
d1
d3
d5
d8
ESC
Oct4
Oct6

Supplementary Figure 7. Chromatin accessibility at sites containing *SoxOct* motifs, Related to Figure 4

(A) Heatmaps of ATAC-seq signal in indicated conditions over the Oct4 bound ChIP-seq peak locations that overlapped with the CO (close-to-open in MEFs to ESCs) category (defined in GSE93029[1]) and further separated into peaks with *SoxOct* motif (upper panel) and without *SoxOct* motif (lower panel). The ATAC signal in MEFs, ESCs, 1F (O, S, K) and 2F (OS, OK, SK) cocktails are from GSE93029. Genomic locations are provided in Supplementary Data 2.

(B) ATAC-seq signal boxplot in Oct4-SK, Oct6-SK and $Oct4^{defSox2}$-SK conditions at days 1 and 5 for CO sites with or without *SoxOct* motif.

(C-D) ATAC-seq signal at day 1 are shown as boxplot from one factor (C) or two factors (D) cocktails over the loci defined in A. The ATAC-seq signal for one factor (in O, S, K) and two factors (in OS, OK, SK) cocktails are from GSE93029.

(E) Oct4-SK and Oct6-SK ATAC-seq signal boxplots for *SoxOct* sites from (A) which intersect with mouse ESC enhancers as defined in[5] (n = 202).

(F) ATAC-seq signal tracks for representative locations with or without *SoxOct* motifs. Genomic coordinates for the summits are listed in Supplementary Table 4.

(G) RNA-seq expression (mean normalized tag counts) for genes associated with the ChIP-seq peaks in (F). RNAseq replicates (n = 2, technical replicates) are shown as individual data points. The boxplots for ATAC-seq signals are normalized using EASeq (DNA fragments per kilobase pairs (kbp) per million (M) reads). P-values in (B-D) were calculated by pairwise comparisons with the unpaired Wilcoxon rank sum test (R function pairwise.wilcox.test) and adjusted for multiple testing using the Holm method. For the boxplots, the midline indicates the median, boxes indicate the upper and lower quartiles and the whiskers indicate 1.5 times interquartile range.

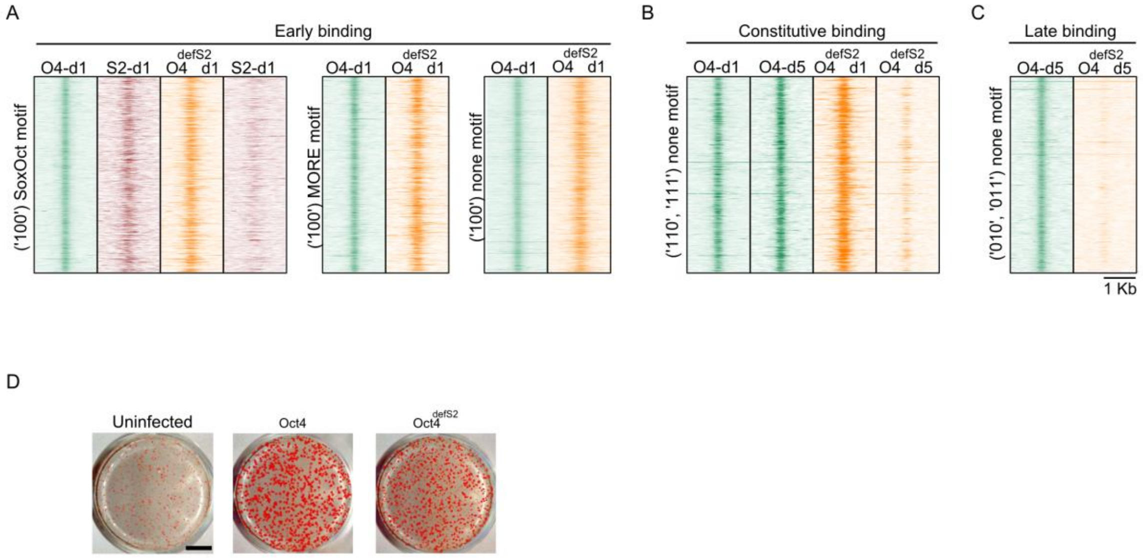
A
Early binding
O4-d1
S2-d1
O4defS2 d1
S2-d1
('100') SoxOct motif
('100') MORE motif
('100') none motif
B
Constitutive binding
O4-d1
O4-d5
O4defS2 d1
O4defS2 d5
('110', '111') none motif
C
Late binding
O4-d5
O4defS2 d5
('010', '011') none motif
1 Kb
D
Uninfected
Oct4
Oct4defS2

Supplementary Figure 8. Oct4^{defSox2} mirrors Oct4 binding at day 1 but not at day 5, Related to Figure 5

(A) Oct4, Oct4^{defSox2} and corresponding Sox2 ChIP-seq signals for the Oct4 occupancy trajectories separated for subsets containing matches to the *SoxOct* motif (left panel), *MORE* motif (middle panel) or lacking either motif ('none', right panel).

(B-C) As in (A) for locations lacking any POU motif ('none') in the constitutively bound ('110' and '111') trajectories (B) or late bound ('010' and '011') trajectories (C).

ChIP-seq signal are displayed with a window of 2 kb centered on the motifs (A-C).

(D) Complementation assay showing whole well images of ZHBTc4[6] ESCs stained with alkaline phosphatase after culturing for 6 days in the presence of Dox to suppress transgenic *Oct4* and transduced with lentiviruses expressing Oct4, Oct4^{defSox2} and an un-transduced control (passage 1); scale 5mm.

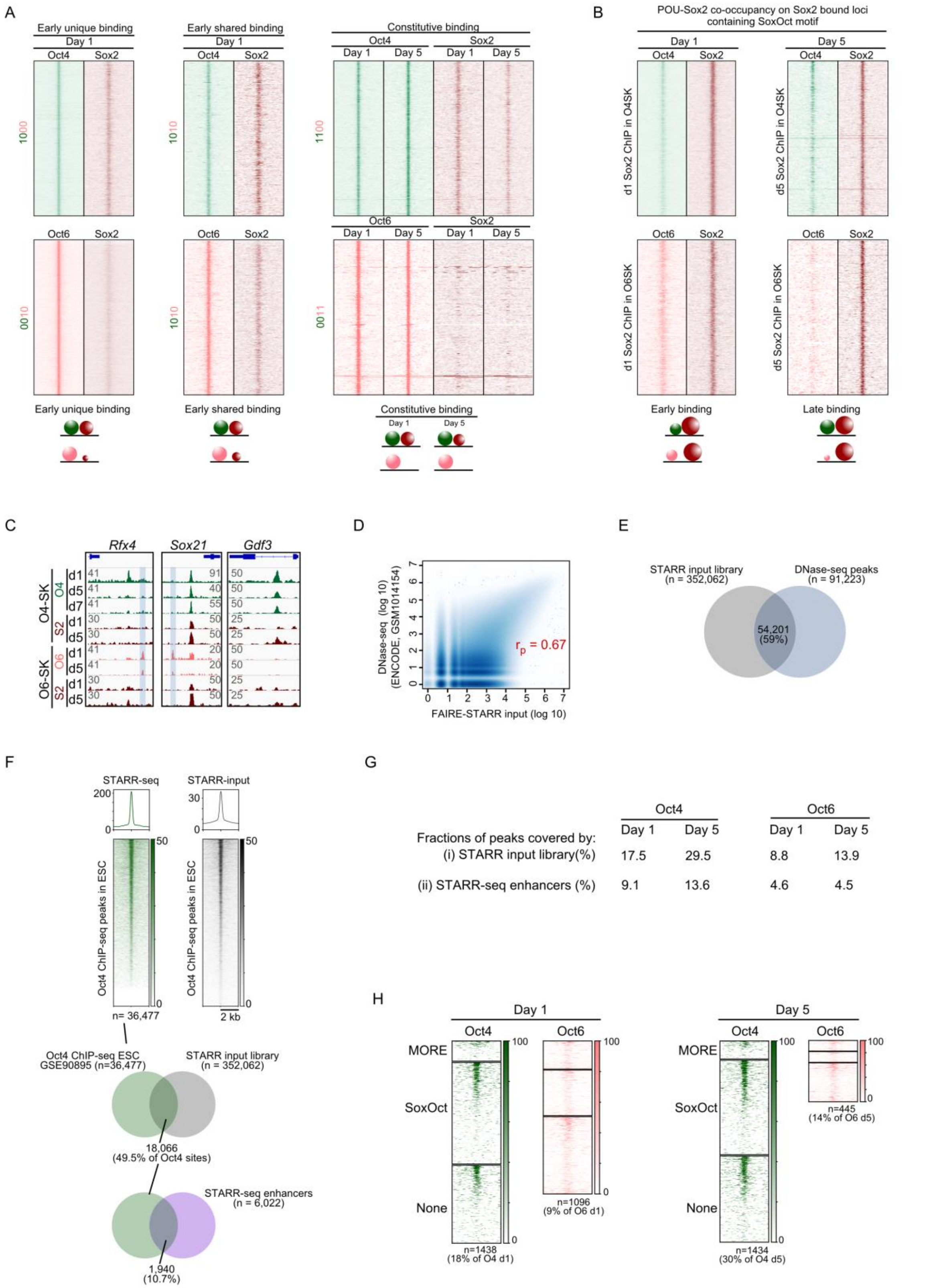
A
Early unique binding
Day 1
Oct4
Sox2
1000
Oct6
0010
Early shared binding
1010
Constitutive binding
Day 5
1100
0011
B
POU-Sox2 co-occupancy on Sox2 bound loci containing SoxOct motif
d1 Sox2 ChIP in O4SK
d5 Sox2 ChIP in O4SK
d1 Sox2 ChIP in O6SK
d5 Sox2 ChIP in O6SK
Early binding
Late binding
C
Rfx4
Sox21
Gdf3
O4-SK
O6-SK
O4
S2
O6
d1
d5
d7
D
DNase-seq (log 10) (ENCODE, GSM1014154)
FAIRE-STARR input (log 10)
r_p = 0.67
E
STARR input library (n = 352,062)
DNase-seq peaks (n = 91,223)
54,201 (59%)
F
STARR-seq
STARR-input
Oct4 ChIP-seq peaks in ESC
n= 36,477
2 kb
Oct4 ChIP-seq ESC GSE90895 (n=36,477)
STARR input library (n = 352,062)
18,066 (49.5% of Oct4 sites)
STARR-seq enhancers (n = 6,022)
1,940 (10.7%)
G
Fractions of peaks covered by:
(i) STARR input library(%)
(ii) STARR-seq enhancers (%)
Oct4
Oct6
Day 1
Day 5
17.5
29.5
8.8
13.9
9.1
13.6
4.6
4.5
H
Day 1
Day 5
MORE
SoxOct
None
n=1438 (18% of O4 d1)
n=1096 (9% of O6 d1)
n=1434 (30% of O4 d5)
n=445 (14% of O6 d5)

Supplementary Figure 9. Comparison of binding patterns and enhancer association of Oct4 and Oct6, Related to Figure 6

(A) ChIP-seq signals for Oct4 (top) and Oct6 (bottom) and with corresponding Sox2 signals. Rows are the indicated occupancy groups and represent sets that are exclusive for Oct4 or Oct6 (Oct4 early:'1000' and Oct4 constitutive: '1100'; Oct6 early:'0010' and Oct6 constitutive: '0011') or shared (Oct4/Oct6 early shared: '1010', middle panel).

(B) ChIP-seq signal heat maps of Oct4 (top), Oct6 (bottom) and corresponding Sox2 occupancy at days 1 (left) and 5 (right). Rows are Sox2 ChIP-seq peaks containing *SoxOct* motifs called in Oct4 (top) or Oct6 (bottom) conditions.

(C) Genome browser tracks showing Oct4, Oct6 and respective Sox2 ChIP-seq signals.

Heatmaps in (A) and (B) show normalised ChIP-seq signals in a 5 kb window centered at peak summits or midpoints of merged occupancy trajectories. Genomic coordinates for the summits are given in Supplementary Table 4.

(D) Scatter plot showing the correlation of FAIRE-STARR-input (Formaldehyde-Assisted Isolation of Regulatory Elements-Self-transcribing active regulatory region sequencing) library and DNase-seq (DNase I hypersensitive sites sequencing, GSM1014154[7]) from E14 mESC. The Pearson's correlation coefficient is indicated.

(E) Venn representing the intersection of STARR input library and DNase-Seq peaks downloaded from GSM1014154[7]) from E14 mESC.

(F) Heatmaps showing STARR-seq and STARR-input library signal on Oct4 bound ChIP-seq peaks in ESCs (GSE90895[4]). The Venn shows the intersection of Oct4 ChIP-seq peaks with STARR-input library (middle panel) and with STARR-seq enhancers (lower panel).

(G) Fraction of peaks from Oct4 and Oct6 ChIP-seq at days 1 and 5 as present in STARR-input and STARR-seq enhancers are indicated in percentages.

(H) Heatmaps showing the STARR-seq signal at the Oct4 and Oct6 ChIP-seq peaks at days 1 and 5 containing matches to *SoxOct*, *MORE* or to none of the two POU motifs ('none'). STARR-seq enhancer signals were normalized with the STARR-input library prepared using accessible regions in ESCs generated by FAIRE[8]. Number and fraction of ChIP-seq peaks overlapping with the STARR input library are indicated underneath the heatmaps.

Supplemental tables

Supplementary Table 1: RNA-seq expression data from ESCs or iPSCs from various labs used as reference; related to Supplementary Figure 1D

ES cells	GEO ID	Culture medium	Reference PMID
OG2-ESC<br>OG2-ESC<br>OG2-iPSC<br>OG2-iPSC<br>OG2-MEF<br>OG2-MEF	GSM2442725<br>GSM2442726<br>GSM2442727<br>GSM2442728<br>GSM2442729<br>GSM2442730	Feeder-free + mES medium + N2B27-2i + 50% KO-DMEM +LIF	Pei Lab[1]
miPSC	GSM2645755	Gelatin + mES medium + 15% Serum/LIF or KOSR	Loh Lab[9]
mESC v6.5	GSM521650	Feeder and Gelatin + mES medium + 15% serum/LIF (No feeder for RNAseq)	Regev lab[10]

Supplementary Table 2: The statistics of ChIP-seq and ATAC-seq libraries, related to Figures 2-6.

ChIP	Sample	Day	Total reads	Mapped deduplicated reads	Peaks
NA	Input	Day1	38,571,244	27,261,634	NA
		Day5	55,348,888	39,444,104	NA
		Day7	10,673,894	5,391,334	NA
POU	Oct4-SK	Day1	145,752,200	103,162,315	8,216
		Day5	53,766,238	35,730,624	4,854
		Day7	37,861,452	27,801,082	3,638
	Oct6-SK	Day1	20,242,068	11,023,192	12,421
		Day5	8,931,144	6,061,399	3,201
	$Oct4^{defSox2}$-SK	Day1	61,519,736	29,364,810	31,070
		Day5	57,585,040	37,885,413	1,038
	Oct4-SM	Day1	17,476,923	8,896,050	101,170
	Oct6-MS	Day1	38,503,128	27,947,258	22,804
Sox2	Oct4-SK	Day1	34,827,014	16,460,243	15,222
		Day5	41,657,078	26,819,329	5,832
	Oct6-SK	Day1	42,255,790	27,743,698	3,242
		Day5	44,787,304	22,851,318	2,673
	$Oct4^{defSox2}$-SK	Day1	40,845,998	25,629,308	5,794
		Day5	46,145,438	23,797,151	21,563
ATAC	Sample	Day	Total reads (millions)	Mapped deduplicated reads	Peaks
Rep 1	Oct4-SK	Day1	75.9M	34,580,294	106,506
		Day5	84.2M	45,677,945	108,631
	Oct6-SK	Day1	80.3M	33,337,802	127,909
		Day5	79.8M	39,431,759	109,793
	$Oct4^{defSox2}$-SK	Day1	79.5M	28,032,791	112,635
		Day5	77.3M	41,912,868	118,955
Rep 2	Oct4-SK	Day1	76.1M	32,409,546	100,393
		Day5	82.2M	41,715,636	119,178
	Oct6-SK	Day1	93.5M	35,909,929	138,081
		Day5	76.0M	36,836,777	103,663
	$Oct4^{defSox2}$-SK	Day1	80.6M	25,920,426	101,589
		Day5	81.5M	42,887,789	123,920

Supplementary Table 3: ChIP-qPCR primers; related to Supplementary Figure 3B

Primer name	Sequence 5'→3'
ChIP-qPCR_Chen3_F	AGTGGGCGGCTTGAGGGCTCAGTCTAAAT
ChIP-qPCR_Chen3_R	CTCTTCCTACCGTGTGGGCTTCAGGGATGC
ChIP-qPCR_Chen4_F	ACTTTCCTCAGGGTTCCCTTGGTGCG
ChIP-qPCR_Chen4_R	TCAGGCAATTCACGGGAATCGGTTATCATA
ChIP-qPCR_Chen43_F	GCAATGGTTAAGCGAGGTTACAGGAACACA
ChIP-qPCR_Chen43_R	AACCCTAAGGCCAGGATGGTCAGTAG
ChIP-qPCR_Chen44_F	GCCATTGTGATGCATATAGGATTATTCACG
ChIP-qPCR_Chen44_R	AACATATACATTTTATACTGTCCATTGGCT
ChIP-qPCR_Chen45_F	TGTAGCCAGCAGACAGGACAAATATCCCTT
ChIP-qPCR_Chen45_R	CCAAGACCACCAAAGTTCCACCTACCCTC
ChIP-qPCR_Chen46_F	GGGGTGGGACAGTGACATCTAAATGACAAT
ChIP-qPCR_Chen46_R	ATGGCCTCTGTTTCATCTTGGTCTGAC
ChIP-qPCR_Chen47_F	CTCATGAGGGCTGCCAGATCACAACA
ChIP-qPCR_Chen47_R	CCTGGGAAGGACAAAAGGAGAGGCTAAACT

Supplementary Table 4: Genome browser coordinates; related figures are mentioned in separate column

Nearest Gene	Chr	Start	End	Peak-ID	Figure
Tet2	chr3	133544389	133544390	oct4WT_O4SK_d1_peak_5219	Fig. 2F, 5D
Col8a1	chr16	57736605	57736606	oct4WT_O4SK_d1_peak_3199a	Fig. 2F
Svil	chr18	5133709	5133710	oct4WT_O4SK_d1_peak_3593	Fig. 2F
Ptprt	chr2	162356333	162356334	oct4WT_O4SK_d5_peak_2796	Fig. 2F
Nfkbia	chr12	55484545	55484546	oct4WT_O4SK_d5_peak_1017	Fig. 2F
Aoah	chr13	20936246	20936247	oct4WT_O4SK_d1_peak_1983	Fig. 2F
Zyg11a	chr4	108182510	108182511	oct4WT_O4SK_d7_peak_2521	Fig. 2F
Sox21	chr14	118230387	118230388	oct4WT_O4SK_d1_peak_2692	Fig. 2G, 5D
Spata13	chr14	60653441	60653442	oct4WT_O4SK_d1_peak_2507	Fig. 2G
Sox7	chr14	63929657	63929658	oct4SM_SMSK_d1_peak_28908	Sup. Fig. 3F
Sox17	chr1	4491943	4491944	oct6MS_MSSK_d1_peak_11a	Sup. Fig. 3F
Pou3f1	chr4	124657948	124657949	oct6MS_MSSK_d1_peak_15552a	Sup. Fig. 3F
Cnpy1	chr5	28210052	28210053	oct4SM_SMSK_d1_peak_69538	Sup. Fig. 3F
Sox2	chr3	34650834	34650835	oct6MS_MSSK_d1_peak_13474	Sup. Fig. 3F
Sox13	chr1	133397732	133397733	oct4SM_SMSK_d1_peak_5058	Sup. Fig. 3F
Kdm2b	chr5	122900391	122900392	oct4SM_SMSK_d1_peak_73063	Sup. Fig. 3F
Runx1t1	chr4	13804005	13804006	oct4WT_O4SK_d1_peak_5375	Sup. Fig. 6E
Hdac4	chr1	91993982	91993983	oct4WT_O4SK_d1_peak_338	Sup. Fig. 6E
Tenm4	chr7	96723241	96723242	oct4YR_YRSK_d1_peak_25198	Sup. Fig. 6E
Sox13	chr1	133397766	133397767	oct4WT_O4SK_d1_peak_443	Sup. Fig. 6E
Ar	chrX	98317702	98317703	oct4WT_O4SK_d1_peak_7888	Sup. Fig. 6E
Bach2/ Map3k7	chr4	32176611	32176612	oct4YR_YRSK_d1_peak_19873	Sup. Fig. 6E
Cnpy1	chr5	28210062	28210063	oct4WT_O4SK_d1_peak_5931	Fig. 5D, Sup Fig. 7G
Pipox	chr11	77894068	77894069	oct4WT_O4SK_d1_peak_1410	Fig. 5D, Sup Fig. 7G
Gdf3	chr6	122608615	122608616	oct4WT_O4SK_d1_peak_6708	Fig. 5D, Sup Fig. 7G
Zmiz2	chr11	6389303	6389304	oct4WT_O4SK_d1_peak_1156	Fig. 5D, Sup Fig. 7G
Gadd45g	chr13	51846606	51846607	oct4WT_O4SK_d1_peak_2115	Fig. 5D
Kdm2b	chr5	122900401	122900402	oct4WT_O4SK_d1_peak_6204	Fig. 5D
Rfx4/Ric8b	chr10	84913371	84913372	oct6WT_O6SK_d1_peak_1436	Sup. Fig. 9C
Sox21	chr14	118226221	118226222	oct6WT_O6SK_d1_peak_4001	Sup. Fig. 9C
Pou3f1	chr4	124658719	124658720	oct6WT_O6SK_d1_peak_8933c	Sup. Fig. 9C

Supplementary Table 5: EMSA probes; related to Figure 2I

Probe name	Sequence 5’→3’
MORE-Spata13-CY5	AGGGT**ATGCATATGAAT**ACAGA
SoxOct-Sox21-Cy5	GTAGATTG**CATTCTAATGCTAAT**CTAGCGT

Supplementary Table 6: cDNA synthesis primers used for reporter assay; related to Figure 2J

Primer name	Sequence 5’→3’
GFP	TGTGTCCCAGAATGTTGCCATCTTCC
Rpl19	AAGACTGATCCACATGAGGCCACAGC

Supplementary Table 7: qPCR primers used for reporter assay; related to Figure 2J

Primer name	Sequence 5’→3’
GFP_F	GGCCAGCTGTTGGGGTGTC
GFP_R	TGCATCACCTTCACCCTCTC
Rpl19_F	ATGTATCACAGCCTGTACCTG
Rpl19_R	CCGCTATGTACAGACACGAG

Supplementary Table 8: Reporter assay sequences used for ESCs transfection; related to Figure 2J

Plasmid name	Sequence 5’→3’	Genomic location	Description	Generated by
Negative control 1	CAGCGAAAGAACATCCTGTGCCCGTCGCT	hg19; chr6: 35,699,789 - 35,699,815	GR binding side	gBlock (IDT)
Negative control 2	CAGCGAAAGAAACTCCGTTGCCCGTCGCT	NA	GR binding site with scrambled GR recognition motif	gBlock (IDT)

Negative control 3	CAGCGAAAGAACAAAATGTTCTCGTCGCT	NA	synthetic palindromic GR binding site	gBlock (IDT)
Positive control (CMV enh)	TCAATATTGGCCATTAGCCATATTATTCAT<br>TGGTTATATAGCATAAATCAATATTGGCTA<br>TTGGCCATTGCATACGTTGTATCTATATCA<br>TAATATGTACATTTATATTGGCTCATGTCC<br>AATATGACCGCCATGTTGGCATTGATTATT<br>GACTAGTTATTAATAGTAATCAATTACGGG<br>GTCATTAGTTCATAGCCCATATATGGAGTT<br>CCGCGTTACATAACTTACGGTAAATGGCCC<br>GCCTGGCTGACCGCCCAACGACCCCCGCCC<br>ATTGACGTCAATAATGACGTATGTTCCCAT<br>AGTAACGCCAATAGGGACTTTCCATTGACG<br>TCAATGGGTGGAGTATTTACGGTAAACTGC<br>CCACTTGGCAGTACATCAAGTGTATCATAT<br>GCCAAGTCCGCCCCCTATTGACGTCAATGA<br>CGGTAAATGGCCCGCCTGGCATTATGCCCA<br>GTACATGACCTTACGGGACTTTCCTACTTG<br>GCAGTACATCTACGTATTAGTCATCGCTAT<br>TACCATGGTGATGCGGTTTTGGCAGTACAC<br>CAATGGGCGTGGATAGCGGTTTGACTCAC<br>GGGGATTTCCAAGTCTCCACCCCATTGACG<br>TCAATGGGAGTTTGTTTTGGCACCAAAATC<br>AACGGGACTTTCCAAAATGTCGTAACAACT	NA	viral enhancer of transcription	gBlock (IDT)
Sox21	GTGTGAGTGAGTGTTTAAAGAATATATATA<br>AAAAGTTCCCCCACTCATTTACATAGCTTC<br>ATCCTTACCTTTCCCATTCAACCCTAGCAA<br>AAAGCATATCTATTCAAAGCCCTTGCAAAA<br>AGCATATCCATTCAAAGGGCATTTAGTCCA<br>TTTCTTTCTTGGCGGGTAAACCTATTCATC<br>AATTGTTCTGCCTTGTAGATTG**CATTCTAA**<br>**TGCTAAT**CTAGCGTGTTAAATATCTTTTTG<br>TTCCCCTTTCACCGCTTTGTCATTCAGTTTA<br>TCCAGTTTTGGTCACCCATTTTATTTATTTA<br>TGCGCCTGCTCCTATTCAGGGG	mm10; chr14: 118,629,364 - 118,629,687	genomic region near Sox21 gene, constitutively bound by Oct4 encompassing a *SoxOct* motif	nested PCR
Sox21 mut	GTGTGAGTGAGTGTTTAAAGAATATATATA<br>AAAAGTTCCCCCACTCATTTACATAGCTTC<br>ATCCTTACCTTTCCCATTCAACCCTAGCAA<br>AAAGCATATCTATTCAAAGCCCTTGCAAAA<br>AGCATATCCATTCAAAGGGCATTTAGTCCA<br>TTTCTTTCTTGGCGGGTAAACCTATTCATC<br>AATTGTTCTGCCTTGTAGATTG**CATTCTAC**<br>**CCGTAGT**CTAGCGTGTTAAATATCTTTTTG<br>TTCCCCTTTCACCGCTTTGTCATTCAGTTTA<br>TCCAGTTTTGGTCACCCATTTTATTTATTTA	NA	genomic region near Sox21 gene, constitutively bound by Oct4 encompassing a	side directed mutagenesis of Sox21 plasmid

	TGCGCCTGCTCCTATTCAGGGG		mutated *SoxOct* motif	
Spata13	CTAAAGTCATAAGGATGATTGGCCTCTGGT TGTTGGGGTTGGCTGTTCGAGACACTGAGG GTGTCAGCAGGCTGAACTTGAACTTGTGAC GTTCATGCTTTAGCTTCCTGAACAGTGGGA TCCAGGTGTGGCTCACCTTGCCCCAGCTGC TGCGTTTTCAGCTTGGGTAGTGTCTTTCCCT GCTTTTGTTGTCAGGGT**ATGCATATGAAT** ACAGATCTGAAGAGCAGATGTCCATGACT TGTGCATATGGATATGCATACCATGACTGT TATTTTAAACATGTGCCTAAGTATAGTGGA GAGTGGGACAGGAGGCAGCGTCCTGGGAT CCAACTCTGGCTGTTTGGAAATGAGCTGAG TGCTCTGTTCTGAGGCCTTTTTGTCGTCCCC CCCCCATATAATATAGGGTTAGTTTAAGCC AAGGAATAAAAGTATAG	Mm10; chr14: 61,272,074 - 61,272,509	genomic region near Spata13 gene, constitutively bound by Oct4 encompassing a *MORE* motif	nested PCR
Spata13 mut	CTAAAGTCATAAGGATGATTGGCCTCTGGT TGTTGGGGTTGGCTGTTCGAGACACTGAGG GTGTCAGCAGGCTGAACTTGAACTTGTGAC GTTCATGCTTTAGCTTCCTGAACAGTGGGA TCCAGGTGTGGCTCACCTTGCCCCAGCTGC TGCGTTTTCAGCTTGGGTAGTGTCTTTCCCT GCTTTTGTTGTCAGGGT**ATCCAGCTGACG** ACAGATCTGAAGAGCAGATGTCCATGACT TGTGCATATGGATATGCATACCATGACTGT TATTTTAAACATGTGCCTAAGTATAGTGGA GAGTGGGACAGGAGGCAGCGTCCTGGGAT CCAACTCTGGCTGTTTGGAAATGAGCTGAG TGCTCTGTTCTGAGGCCTTTTTGTCGTCCCC CCCCCATATAATATAGGGTTAGTTTAAGCC AAGGAATAAAAGTATAG	NA	genomic region near Spata13 gene, constitutively bound by Oct4 encompassing a mutated *MORE* motif	side directed mutagenesis of Spata13 plasmid

Supplementary Table 9: qPCR primers used for the detection of endogenous Oct4; related to Supplementary Figure 4A

Primer name	Sequence 5'→3'
endo-mOct4-RT-F	TAGGTGAGCCGTCTTTCCAC
endo-mOct4-RT-R	GCTTAGCCAGGTTCGAGGAT
mActin-RT-F	TGCTAGGAGCCAGAGCAGTA
mActin-RT-R	AGTGTGACGTTGACATCCGT
mGAPDH-RT-F	AACTTTGGCATTGTGGAAGGGCTCA
mGAPDH-RT-R	TTGGCAGCACCAGTGGATGCAGGGA

Supplementary Table 10: qPCR primers used for marker gene quantification in ZHBTc4 ES cells; related to Figure 5G

Primer name	Sequence 5'→3'
Oct4-CDS-F	GGCTAGAGAAGGATGTGGTTCGAG
Oct4-CDS-R	CCTGGGAAAGGTGTCCCTGTAG
Sox2-F	ACGGCCATTAACGGCACACT
Sox2-R	TTTTGCACCCCTCCCAATTC
Nanog-F	CTTTCACCTATTAAGGTGCTTGC
Nanog-R	ATGGCATCGGTTCATCATGGTAC
Esrrb-F	GCCTTTACTATCTGTGCCTGGT
Esrrb-R	TAGTGCTTCTCTTTGGTGCTGT
Rex1-F	GGCTGCGAGAAGAGCTTTATTCA
Rex1-R	AGCATTTCTTCCCGGCCTTT
Cdx2-F	ACCGGAATTGTTTGCTGCTGT
Cdx2-R	TCCCGACTTCCCTTCACCAT
Rpl37a-F	ACTTGCTCCTTCTGTGGCAAGAC
Rpl37a-R	TTCATGCAGGAACCACAGTGC

Supplementary Table 11: Antibodies used in the study

Antibodies	Source	Identifier	Application and usage
Oct-3/4 (N-19) X	Santa Cruz Biotechnology	Sc-8628 X	ChIP-seq (Total 10μg, 1:20), Western Blot (1:5000)
Oct-6 (C-20) X	Santa Cruz Biotechnology	Sc-11661 X	ChIP-seq (Total 10μg, 1:20), Western Blot (1:5000)
Sox-2 Antibody (Y-17) X	Santa Cruz Biotechnology	sc-17320 X	ChIP-seq (Total 10μg, 1:20)
Sox2 Antibody	Cell Signaling Technology	2748s	ChIP-seq (Total 10μg, 1:1)
Actin (I-19)-R	Santa Cruz Biotechnology	Sc-1616-R	Western Blot (1:500)
IRDye® 800CW Donkey anti-Goat IgG (H + L)	LI-COR	926-32214	Western Blot (1:10,000)
IRDye® 680RD Donkey anti-Rabbit IgG (H + L)	LI-COR	926-68073	Western Blot (1:10,000)
Cdx2	New England Biolabs	3977S	ICC (1:1000)
anti-Nanog	eBioscience	eBioMLC-51	ICC (1:500, Figure 5F)
anti-Oct4	Santa Cruz Biotechnology	sc-8628	ICC (1:500, Figure 5F)
anti-Nanog	Novus Biologicals	NB100-58842	ICC (1:500, Figure 1B, Sup. Fig. 1B)
NucBlue Fixed Cell Stain (DAPI)	Thermo Fisher Scientific	R37606	ICC (1:50, Sup. Fig. 1B)
Anti-Cdh1 (ANTI−CD324 DECMA1 EF660)	Thermo Fisher Scientific	50−3249−82	FACS (1:40)
Mouse IgG1, κ Isotype Control	BD biosciences	555746	FACS (1:10)
DAPI	BD biosciences	564907	FACS (1:1000)

Supplementary references

1 Li, D. *et al.* Chromatin Accessibility Dynamics during iPSC Reprogramming. *Cell stem cell* 21, 819-833 e816, doi:10.1016/j.stem.2017.10.012 (2017).

2 Chen, X. *et al.* Integration of external signaling pathways with the core transcriptional network in embryonic stem cells. *Cell* 133, 1106-1117, doi:10.1016/j.cell.2008.04.043 (2008).

3 Knaupp, A. S. *et al.* Transient and Permanent Reconfiguration of Chromatin and Transcription Factor Occupancy Drive Reprogramming. *Cell stem cell* 21, 834-845 e836, doi:10.1016/j.stem.2017.11.007 (2017).

4 Chronis, C. *et al.* Cooperative Binding of Transcription Factors Orchestrates Reprogramming. *Cell* 168, 442-459 e420, doi:10.1016/j.cell.2016.12.016 (2017).

5 Shen, Y. *et al.* A map of the cis-regulatory sequences in the mouse genome. *Nature* 488, 116-120, doi:10.1038/nature11243 (2012).

6 Niwa, H., Miyazaki, J. & Smith, A. G. Quantitative expression of Oct-3/4 defines differentiation, dedifferentiation or self-renewal of ES cells. *Nature genetics* 24, 372-376, doi:10.1038/74199 (2000).

7 Yue, F. *et al.* A comparative encyclopedia of DNA elements in the mouse genome. *Nature* 515, 355-364, doi:10.1038/nature13992 (2014).

8 Giresi, P. G., Kim, J., McDaniell, R. M., Iyer, V. R. & Lieb, J. D. FAIRE (Formaldehyde-Assisted Isolation of Regulatory Elements) isolates active regulatory elements from human chromatin. *Genome research* 17, 877-885, doi:10.1101/gr.5533506 (2007).

9 Fang, H. T. *et al.* Global H3.3 dynamic deposition defines its bimodal role in cell fate transition. *Nature communications* 9, 1537, doi:10.1038/s41467-018-03904-7 (2018).

10 Guttman, M. *et al.* Ab initio reconstruction of cell type-specific transcriptomes in mouse reveals the conserved multi-exonic structure of lincRNAs. *Nature biotechnology* 28, 503-510, doi:10.1038/nbt.1633 (2010).
