# Supplementary material for: Pluripotency reprogramming by competent and incompetent POU factors uncovers temporal dependency for Oct4 and Sox2
Source: Nat Commun. 2019 Aug 2;10:3477. doi: 10.1038/s41467-019-11054-7 (PMC6677745; doi:10.1038/s41467-019-11054-7)
Supplement: Supplementary file 3 — Reporting Summary [file 41467_2019_11054_MOESM3_ESM.pdf]

nature research

Corresponding author(s): Ralf Jauch

Reporting Summary

Nature Research wishes to improve the reproducibility of the work that we publish. This form provides structure for consistency and transparency in reporting. For further information on Nature Research policies, see Authors & Referees and the Editorial Policy Checklist.

Statistical parameters

When statistical analyses are reported, confirm that the following items are present in the relevant location (e.g. figure legend, table legend, main text, or Methods section).

n/a	Confirmed	
☐	☒	The exact sample size (*n*) for each experimental group/condition, given as a discrete number and unit of measurement
☐	☒	An indication of whether measurements were taken from distinct samples or whether the same sample was measured repeatedly
☐	☒	The statistical test(s) used AND whether they are one- or two-sided<br>*Only common tests should be described solely by name; describe more complex techniques in the Methods section.*
☒	☐	A description of all covariates tested
☐	☒	A description of any assumptions or corrections, such as tests of normality and adjustment for multiple comparisons
☐	☒	A full description of the statistics including central tendency (e.g. means) or other basic estimates (e.g. regression coefficient) AND variation (e.g. standard deviation) or associated estimates of uncertainty (e.g. confidence intervals)
☐	☒	For null hypothesis testing, the test statistic (e.g. *F*, *t*, *r*) with confidence intervals, effect sizes, degrees of freedom and *P* value noted<br>*Give P values as exact values whenever suitable.*
☒	☐	For Bayesian analysis, information on the choice of priors and Markov chain Monte Carlo settings
☒	☐	For hierarchical and complex designs, identification of the appropriate level for tests and full reporting of outcomes
☒	☐	Estimates of effect sizes (e.g. Cohen's *d*, Pearson's *r*), indicating how they were calculated
☐	☒	Clearly defined error bars<br>*State explicitly what error bars represent (e.g. SD, SE, CI)*

Our web collection on statistics for biologists may be useful.

Software and code

Policy information about availability of computer code

Data collection

Image Studio™ Lite (Western blot images and band quantification),
FlowJo V10 (FACS)

Data analysis

The main command-line codes used to analyze RNA-seq, ATAC-seq and ChIP-seq experiments are described in methods section.
Bowtie v2.3.4
Samtools v1.3.1
Bedtools v2.27.1
MACS v2
IGV v2.3.92
EAseq V1.04 (For ChIP-seq and ATAC-seq related plots)
Chimera v1.11 (structural modeling)
Metascape v3.0
HOMER v4.10
glbase
Inkscape v0.48.5 (Vector graphics drawing tool)
deepTools
UMI-tools

R studio and following R packages:

Data.table
DEseq2 v1.20.0
ggplot2/ggrepel
Tidyr/data wrangling
stringi/stringr
Pretty heatmap
EDAseq v2.4.1
Bioconductor

For manuscripts utilizing custom algorithms or software that are central to the research but not yet described in published literature, software must be made available to editors/reviewers upon request. We strongly encourage code deposition in a community repository (e.g. GitHub). See the Nature Research guidelines for submitting code & software for further information.

Data

Policy information about availability of data

All manuscripts must include a data availability statement. This statement should provide the following information, where applicable:

- Accession codes, unique identifiers, or web links for publicly available datasets
- A list of figures that have associated raw data
- A description of any restrictions on data availability

The accession number for the ChIP-seq, RNA-seq, and ATAC-seq data reported in this paper is NCBI GEO: GSE103980. The authors declare that the data supporting the findings of this study are available within the article and its supplementary information files, or from the corresponding author upon reasonable request.

Field-specific reporting

Please select the best fit for your research. If you are not sure, read the appropriate sections before making your selection.

☒ Life sciences ☐ Behavioural & social sciences ☐ Ecological, evolutionary & environmental sciences

For a reference copy of the document with all sections, see nature.com/authors/policies/ReportingSummary-flat.pdf

Life sciences study design

All studies must disclose on these points even when the disclosure is negative.

Sample size	We did not use any statistical methods to determine sample size and also did not perform any in-vivo analysis. All data was generated using cell-culture experiments. Time course RNA-seq was done in duplicates at days 0, 1, 3, 5, 8 of reprogramming, including MEFs. ATAC-seq experiments were performed in duplicates at days 1 and 5. ChIP-seq was performed once at days 1 and 5 of reprogramming. All the q-PCR based experiment were performed three to four times the exact number of which is mentioned in figure legends or in method section.
Data exclusions	No data was excluded from the analysis.
Replication	All experimental findings were reliably reproduced.
Randomization	The samples were not randomized.
Blinding	Investigators were not blinded to the sample identities.

Reporting for specific materials, systems and methods

Materials & experimental systems

n/a	Involved in the study	
☒	☐	Unique biological materials
☐	☒	Antibodies
☐	☒	Eukaryotic cell lines
☒	☐	Palaeontology
☐	☒	Animals and other organisms
☒	☐	Human research participants

Methods

n/a	Involved in the study	
☐	☒	ChIP-seq
☐	☒	Flow cytometry
☒	☐	MRI-based neuroimaging

Antibodies

Antibodies used	Oct-3/4 (N-19) X (Sc-8628 X) Santa Cruz Biotechnology;<br>Oct-6 (C-20) X (Sc-11661 X) Santa Cruz Biotechnology;<br>Sox-2 Antibody (Y-17) X (sc-17320 X) Santa Cruz Biotechnology;<br>Sox2 Antibody (2748s) Cell Signaling Technology;<br>Actin (I-19)-R (Sc-1616-R) Santa Cruz Biotechnology;<br>IRDye® 800CW Donkey anti-Goat IgG (H + L) (926-32214) LI-COR;<br>IRDye® 680RD Donkey anti-Rabbit IgG (H + L) (926-68073) LI-COR;<br>Cdx2 (3977S) New England Biolabs;<br>anti-Nanog (eBioMLC-51) eBioscience;<br>anti-Oct4 (sc-8628) Santa Cruz Biotechnology;<br>anti-Nanog (NB100-58842)Novus Biologicals;<br>NucBlue Fixed Cell Stain (DAPI)(R37606) Thermo Fisher Scientific;<br>Anti-Cdh1 (ANTI-CD324 DECMA1 EF660) (50-3249-82)Thermo Fisher Scientific;<br>Mouse IgG1, κ Isotype Control (555746) BD biosciences<br>DAPI (564907) BD biosciences<br>The list of antibodies is also summarized in Supplementary Table 12 in supplemental information of the manuscript.
Validation	No new antibodies were generated in this study. All the antibodies used in this study have been validated in numerous studies and by the manufacturers. ChIP-seq antibodies were validated by Western Blot detection according to the manufacturer's instruction.

Eukaryotic cell lines

Policy information about cell lines

Cell line source(s)	Plat-E cells and OG-2 MEF cells were obtained from central core facility of GIBH.
Authentication	No cell line authentication was performed.
Mycoplasma contamination	All cell lines were tested for mycoplasma contamination.
Commonly misidentified lines (See ICLAC register)	No commonly misidentified cell lines were used in this study.

Animals and other organisms

Policy information about studies involving animals; ARRIVE guidelines recommended for reporting animal research

Laboratory animals	OG2-transgenic mouse were purchased from The Jackson Laboratory; ID: B6;CBA-Tg(Pou5f1-EGFP)2Mnn/J; Stock No: 004654/ OG2; Strain: of origin: (CBA/CaJ X C57BL/6J)F2. The OG2-MEFs used in this study were isolated from E13.5 offsprings of these mice.
Wild animals	The study did not involve wild animals
Field-collected samples	The study did not involve samples collected from the field

ChIP-seq

Data deposition

☒ Confirm that both raw and final processed data have been deposited in a public database such as GEO.

☒ Confirm that you have deposited or provided access to graph files (e.g. BED files) for the called peaks.

Data access links<br>*May remain private before publication.*	https://www.ncbi.nlm.nih.gov/geo/query/acc.cgi?acc=GSE103980
Files in database submission	The ChIP-seq peaks, Bigwig and raw fastq files are available for following sample:<br>oct4WT_O4SK_d1 (GSM2787242)<br>oct4WT_O4SK_d5 (GSM2787244)<br>oct4WT_O4SK_d7 (GSM2787245)<br>oct6WT_O4SK_d1 (GSM2787247)<br>oct6WT_O4SK_d5 (GSM2787249)<br>oct4YR_YRSK_d1 (GSM2787255)<br>oct4YR_YRSK_d5 (GSM2787257)<br>Oct4SM_SMSK_d1 (GSM3674279)<br>Oct6MS_MSSK_d1 (GSM3674280)<br>Sox2_O4SK_d1 (GSM3381541)<br>Sox2_O4SK_d5 (GSM3381542)

Sox2_O6SK_d1 (GSM3381543)
Sox2_O6SK_d5 (GSM3381544)
Sox2_YRSK_d1 (GSM3381545)
Sox2_YRSK_d5 (GSM3381546)
InputDay1 (GSM3381547)
InputDay5 (GSM3381548)
InputDay7 (GSM2787266)

Genome browser session (e.g. UCSC)

All the BigWig files have been deposited at GEO. The files could be accessed at: https://www.ncbi.nlm.nih.gov/geo/query/acc.cgi?acc=GSE103978

Methodology

Replicates

The ChIP-seq experiment was done once.

Sequencing depth

ChIP-seq was done with paired end reads of 125 bp (day 7 Oct4-ChIP) or 150 bp for other samples. The details of sequencing depth, uniquely mapped reads and peak numbers is provided in supplementary table S7 in manuscript.

Antibodies

Oct-3/4 (N-19) X (Sc-8628 X), Lot-12415 Santa Cruz Biotechnology;
Oct-6 (C-20) X (Sc-11661 X), Lot-G0606 and G0608 Santa Cruz Biotechnology;
Sox2 Antibody (2748s), Lot-2 Cell Signaling Technology.

Peak calling parameters

Peaks were called using MACS2 with options --call-summits --to-large -p 0.0001 –bdg options. Homer and Dfilter were also tested but the MACS2 peaks were used for subsequent analysis.

Data quality

ChIP-seq peaks were called with a very stringent p-value of 0.001 to 0.0001 (default being 0.05). The numbers of ChIP-seq peaks obtained for each sample are listed in supplementary table S7 in manuscript.

Software

ChIP-seq reads were aligned to the mouse genome (mm10) using Bowtie2 with settings 'end-to-end' and 'very-sensitive'. Samtools was used to discard reads with mapping quality < 10 (samtools view q=10) and only unique reads were kept (samtools rmdup) to account for PCR bias. MACS2 was used for peak calling. EAseq tool was used to generate all the heat-maps and read pileup plots.

Flow Cytometry

Plots

Confirm that:

☒ The axis labels state the marker and fluorochrome used (e.g. CD4-FITC).

☒ The axis scales are clearly visible. Include numbers along axes only for bottom left plot of group (a 'group' is an analysis of identical markers).

☒ All plots are contour plots with outliers or pseudocolor plots.

☒ A numerical value for number of cells or percentage (with statistics) is provided.

Methodology

Sample preparation

MEFs and reprogramming intermediates at days 3, 5 and 8 were dissociated with trypsin. Cells were collected by centrifugation at 300g/5min and kept on ice hereafter. Cells were washed twice with 1mL FACS buffer (1×DPBS, 5% Fetal calf serum, 20 mM HEPES pH7.2-7.5). Cells were incubated in 5% mouse serum containing 100 µl FACS buffer for 10 minutes. Cdh1 antibody (Thermo Fisher Scientific, #50–3249–82) or Mouse IgG1, κ Isotype Control (BD biosciences, #555746) was added directly and incubated for 20-25 mins. Cells were washed twice with FACS buffer. DAPI (BD biosciences, #564907) was used for dead cell exclusion. Cells were passed through 40µm cell strainer (Corning, # CLS431750-50EA) before analysis.

Instrument

BD LSRFortessa

Software

FlowJo V10 (https://www.flowjo.com/)

Cell population abundance

Cell debris were filtered by DAPI positive signal and doublets were excluded by SSCA-FSCW gating.

Gating strategy

To remove the debris and dead cells, initially SSCA versus FSCA gating was used. The live cells were gated by the exclusion of DAPI positive cells. The DAPI negative cells were then gated based on SSCA versus FSCW to remove the doublets. One more round of SSCH versus SSCA was used to further enrich the single cells by this stringent gating. Cells were then subjected to further downstream analysis.

☒ Tick this box to confirm that a figure exemplifying the gating strategy is provided in the Supplementary Information.
