# Supplementary material for: Pluripotency reprogramming by competent and incompetent POU factors uncovers temporal dependency for Oct4 and Sox2
Source: Nat Commun. 2019 Aug 2;10:3477. doi: 10.1038/s41467-019-11054-7 (PMC6677745; doi:10.1038/s41467-019-11054-7)
Supplement: Supplementary file 4 — Description of Additional Supplementary Files [file 41467_2019_11054_MOESM4_ESM.pdf]

Description of Additional Supplementary Files

Supplementary Data 1. This table contains the list of differentially expressed genes in Oct4-SK, $Oct4^{defSox2}$ and Oct6-SK conditions when compared with GFP-SK conditions at days 0, 1, 3, 5 and 8 of reprogramming process. Related to Figures S2D-E.

Supplementary Data 2. This is an excel workbook that contains genomic coordinates of peaks for Figures 2A, 4B, 4D, 4E, 4F, 6A, S4C, S4E, S5E, S5F, S6A, S7A on different excel sheets.
